# Photoreversible interconversion of a phytochrome photosensory module in the crystalline state

**DOI:** 10.1073/pnas.1912041116

**Published:** 2019-12-18

**Authors:** E. Sethe Burgie, Jonathan A. Clinger, Mitchell D. Miller, Aaron S. Brewster, Pierre Aller, Agata Butryn, Franklin D. Fuller, Sheraz Gul, Iris D. Young, Cindy C. Pham, In-Sik Kim, Asmit Bhowmick, Lee J. O’Riordan, Kyle D. Sutherlin, Joshua V. Heinemann, Alexander Batyuk, Roberto Alonso-Mori, Mark S. Hunter, Jason E. Koglin, Junko Yano, Vittal K. Yachandra, Nicholas K. Sauter, Aina E. Cohen, Jan Kern, Allen M. Orville, George N. Phillips, Richard D. Vierstra

**Affiliations:** ^a^Department of Biology, Washington University in St. Louis, St. Louis, MO 63130;; ^b^Department of Biosciences, Rice University, Houston, TX 77005;; ^c^Molecular Biophysics and Integrated Bioimaging Division, Lawrence Berkeley National Laboratory, Berkeley, CA 94720;; ^d^Diamond Light Source Ltd., Didcot, Oxfordshire OX11 0DE, United Kingdom;; ^e^Linac Coherent Light Source, SLAC National Accelerator Laboratory, Menlo Park, CA 94025;; ^f^Stanford Synchrotron Radiation Lightsource, SLAC National Accelerator Laboratory, Menlo Park, CA 94025;; ^g^Research Complex at Harwell, Rutherford Appleton Laboratory, Didcot, Oxfordshire OX11 0DE, United Kingdom;; ^h^Department of Chemistry, Rice University, Houston, TX 77005

**Keywords:** phytochrome, photoreceptor, X-ray crystallography

## Abstract

A major barrier to defining the structural intermediates that arise during the reversible photointerconversion of phytochromes between their biologically inactive and active states has been the lack of crystals that faithfully undergo this transition within the crystal lattice. Here, we describe a crystalline form of the cyclic GMP phosphodiesterases/adenylyl cyclase/FhlA (GAF) domain from the cyanobacteriochrome PixJ in *Thermosynechococcus elongatus* assembled with phycocyanobilin that permits reversible photoconversion between the blue light-absorbing Pb and green light-absorbing Pg states, as well as thermal reversion of Pg back to Pb. The X-ray crystallographic structure of Pb matches previous models, including autocatalytic conversion of phycocyanobilin to phycoviolobilin upon binding and its tandem thioether linkage to the GAF domain. Cryocrystallography at 150 K, which compared diffraction data from a single crystal as Pb or after irradiation with blue light, detected photoconversion product(s) based on F_obs_ − F_obs_ difference maps that were consistent with rotation of the bonds connecting pyrrole rings C and D. Further spectroscopic analyses showed that phycoviolobilin is susceptible to X-ray radiation damage, especially as Pg, during single-crystal X-ray diffraction analyses, which could complicate fine mapping of the various intermediate states. Fortunately, we found that PixJ crystals are amenable to serial femtosecond crystallography (SFX) analyses using X-ray free-electron lasers (XFELs). As proof of principle, we solved by room temperature SFX the GAF domain structure of Pb to 1.55-Å resolution, which was strongly congruent with synchrotron-based models. Analysis of these crystals by SFX should now enable structural characterization of the early events that drive phytochrome photoconversion.

## Significance

A major hurdle in structurally defining the sequence of events that underpin the photointerconversion of phytochromes between their dark-adapted and photoactivated states has been the lack of crystals that undergo these transitions. Here, we describe a crystalline form of the GAF domain from *Thermosynechococcus elongatus* PixJ within the cyanobacteriochrome subfamily that undergoes reversible photointerconversion and thermal reversion back to the dark-adapted state. Preliminary cryocrystallography of irradiated crystals detected movements of the phycoviolobilin chromophore indicative of a D pyrrole ring rotation. However, X-ray hypersensitivity of both absorbing states might complicate interpretation. Fortunately, we found that PixJ is amenable to serial femtosecond X-ray diffraction methods, which we used to generate a 1.55-Å-resolution model of the dark-adapted state at room temperature.

Almost all cellular organisms employ one or more photoreceptors to sense their ambient light environment. One prominent collection within the bacterial, fungal, algal, and plant kingdoms are the phytochromes (Phys), identified as homodimers that harbor a bilin (or open-chain tetrapyrrole) chromophore cradled within a signature cyclic GMP phosphodiesterases/adenylyl cyclases/FhlA (GAF) domain capable of reversible photochromic transitions between 2 stable conformers (1, 2). The most studied Phys photointerconvert between red light-absorbing Pr and far-red light-absorbing Pfr states, with Pr representing the dark-adapted state and Pfr representing the photoactivated and often biologically active state (3). Pfr can also revert back to Pr by a thermal relaxation process whose rate is temperature-dependent, which for some Phys permits thermosensation by effectively competing with photoactivation (4, 5). In addition, novel Phys have evolved to detect light–color pairs other than red/far red, including ultraviolet/blue, blue/green, or blue/orange, or to prefer Pfr as the dark-adapted state and require far-red light for photoconversion to Pr (1, 2).

Regardless of their absorption ranges, photoexcitation of Phys is thought to trigger a reversible 15*Za*⇋15*Ea* isomerization of the bilin. This rotation alters its extensive π-bond conjugation system and thus its light-absorption properties, with the ratio of dark-adapted and photoactivated states then governed by the spectral quality of the light environment (1, 2). Through comparisons of the few available Phys with models of both structural end states (6–11) and cryocrystallography of irradiated crystals by pump-quench and temperature-scan methods (12, 13), a number of conformational changes have been identified that impact the contacts between the bilin and the GAF domain and ultimately the neighboring regions, which could propagate the light signaling after bilin isomerization. Included is a sliding of the bilin within the GAF pocket, adjustment of amino acid contacts with the bilin propionate side chains, and conformational shifts in surrounding motifs, the most prominent being a β-strand–to–α-helical conversion of the hairpin (or tongue) loop emerging from the adjacent Phy-specific (PHY) domain to touch the GAF domain near the chromophore.

While various spectroscopic studies have identified several spectrally distinct intermediates that arise on pico- to millisecond timescales (e.g., refs. 12 and 14–17) and X-ray scattering studies have observed lifetimes of small- and large-scale structural changes along various photoconversion pathways (e.g., refs. 18 and 19), the precise identity and sequence of the structural changes that follow photoexcitation remain unresolved due to a lack of high-resolution structural information from intermediates.

Clearly, time-resolved X-ray crystallography of Phys throughout the photoexcitation reaction cycle would be informative in defining the interconversion sequence. At present, a major hurdle has been the lack of crystal forms that permit this photoconversion in crystallo. Invariably, the crystalline species either fails to fully transition or the crystals shatter or dissolve upon excitation, implying that important conformational changes associated with photoconversion are restricted by crystal packing (1). This is partially because of a focus on Phys containing the hairpin motif, whose large motions and accompanying displacement of the PHY domain have precluded observations on the fully mature, light-activated state (12, 13).

**Fig. 1.**
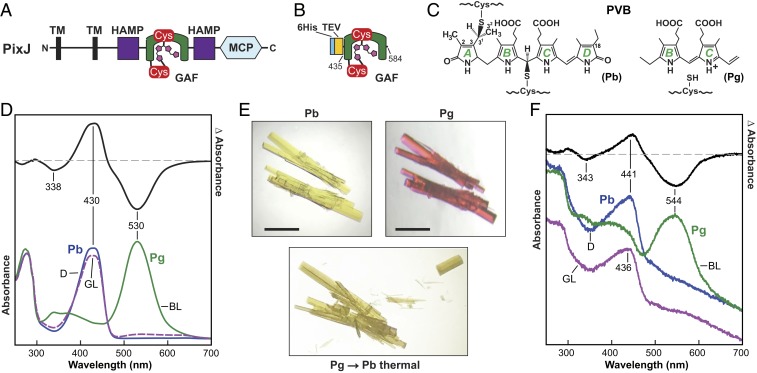
GAF domain from *T. elongatus* PixJ assembled with PCB reversibly photointerconverts between Pb and Pg while in a crystal lattice. (*A*) Domain organization of the full-length (940-residue) PixJ photoreceptor. Amino (N) and carboxyl (C) termini are indicated. PVB with its 2 Cys-mediated thioether linkages as Pb is shown in magenta. HAMP, histidine kinase/adenylyl cyclase/methyl-binding protein/phosphatase; MCP, methyl-accepting chemotaxis protein; TM, transmembrane. (*B*) The GAF domain comprising residues 435 to 584 expressed with a TEV protease-cleavable 6His tag. (*C*) Chemical diagram of PVB that forms upon addition of PCB showing the position of the 2 thioether linkages to the GAF domain. (*C*, *Left*) Complete diagram of Pb. (*C*, *Right*) Partial diagram of Pg showing the region around the B and C pyrrole rings. Numbering of the carbon atoms and labels for the pyrrole rings are included. (*D*) Solution absorption spectra of *Te*PixJ(GAF) at pH 5.5. The absorption spectrum for Pb (blue line) was recorded in dark-adapted samples whereas that for Pg (green line) was recorded after a saturating irradiation with blue light (BL). The purple dashed line identifies the absorption spectrum of the photocycled Pb product generated by irradiating Pg with green light (GL). Dark-adapted Pb-minus-Pg difference spectrum (black line) is shown above at half-magnitude. The wavelengths of difference peak minima and maxima are indicated. (*E*) Orthorhombic crystals of *Te*PixJ(GAF) as Pb (yellow) readily photoconvert at 23 °C to Pg (magenta) using blue light, and then thermally revert back to Pb upon incubation in the dark. (Scale bars, 1 mm.) (*F*) Absorption and difference spectra at 23 °C of *Te*PixJ(GAF) crystals that reversibly photointerconvert between Pb and Pg. The labeling scheme corresponds to *D*. The spectrum for photocycled Pb (Pb→Pg→Pb) is offset for clarity.

In an effort to generate photoconvertible crystals of a Phy, we explored the use of unusual variants within the cyanobacteriochrome (CBCR) subfamily that interconvert between their dark-adapted and activated conformers using just the bilin-occupied GAF domain (2, 20). Of interest was PixJ from the thermophilic cyanobacterium *Thermosynechococcus elongatus* (*Te*), which transitions almost completely between blue light-absorbing Pb and green light-absorbing Pg end states using a phycoviolobilin (PVB) chromophore (Fig. 1 *A*–*C*) (21, 22). In addition to the conventional thioether linkage that connects C522 within the GAF domain to the C3^1^ carbon of the A pyrrole ethylidene side chain, PVB is attached via a second thioether linkage between C494 of the GAF domain and the C10 carbon between the B and C pyrrole rings (6, 21). The end result is conversion of C10 from sp^2^ to sp^3^ hybridization, which shortens the π-conjugation system of PVB and induces the substantial hypsochromic shift from green- to blue-light absorption (6).

Previous X-ray crystallographic analyses of the GAF domain from *Te*PixJ generated atomic models for the Pb and Pg end states (6, 7). Comparisons implied that photoconversion involves isomerization at the C15=C16 bond, a rupture of the C494–C10 thioether linkage, restoration of the π-conjugation between the B and C pyrrole rings, rotation and translation of the bilin in the GAF pocket, and conformational rearrangements of the protein backbone and surrounding amino acid side chains. However, the transitional motions and order of these structural changes remained speculative.

To help unravel this photoconversion sequence, we produced a construction of the GAF domain from *Te*PixJ, hereafter called *Te*PixJ(GAF), that enabled production of crystals that retain reversible photoconversion between Pb and Pg as well as thermal reversion from Pg back to Pb. We then obtained a high-resolution X-ray crystallographic structure of the Pb state and applied cryocrystallography at an elevated temperature to detect a photoconversion product(s) in crystallo. These analyses also revealed that the PVB chromophore in the Pb and Pg states is vulnerable to X-ray–induced alterations of its electronic structure. However, by using serial femtosecond crystallography (SFX) with an X-ray free-electron laser (XFEL) (23, 24), probing Phy photoconversion with minimal radiation damage and at fast timescales should now be possible. As proof of principle, we present a high-resolution atomic model of *Te*PixJ(GAF) acquired using SFX methods for Pb.

## Results

### The GAF Domain of *T. elongatus* PixJ Reversibly Photoconverts in Crystallo.

Our goal was to identify a Phy fragment that photoconverts reversibly both in solution and in crystallo, and that faithfully expresses and crystallizes in quantities sufficient for time-resolved structural studies. We postulated that CBCRs could be ideal sources because their isolated GAF domains are often sufficient for full photoconversion (1, 2). Additionally, CBCRs are often missing the dynamic hairpin motif, indicating the conformational differences between the dark-adapted and photoinduced end states might be sufficiently small to preserve the crystal lattice contacts during photoconversion. Guided by previous crystallographic models of the *Te*PixJ GAF domain (6), we engineered a minimal construction of the photosensory module for recombinant expression (Fig. 1 *A* and *B*). The fragment encompassed residues 435 to 584 and was preceded by a tobacco etch virus (TEV) protease-cleavable, hexahistidine (6His) tag. Because residue 435 is a serine, the final product would be free of any extraneous amino- or carboxyl-terminal residues after trimming with the TEV protease.

Coexpression of the 6His-*Te*PixJ(GAF) polypeptide in *Escherichia coli* with the heme oxygenase and phycocyanobilin reductase enzymes necessary to convert heme to phycocyanobilin (PCB) (20) followed by affinity enrichment and TEV protease cleavage produced a minimal photoactive chromoprotein. It had a dark-adapted Pb absorption indicative of a bilin bound covalently to the apoprotein through thioether linkages to the C3^1^ and C10 carbons (Fig. 1 *C* and *D*). Faithful photointerconversion between Pb and Pg was readily evident upon sequential blue- and green-light irradiations (420 and 530 nm, respectively), verifying the identity of the chromophore as PVB, which necessitated the autocatalytic translation of the C4=C5 bond in PCB to the C2=C3 bond in PVB. Photoconversion in solution was reversible between the Pb and Pg states, and was spectrally indistinguishable over a pH range of 5.5 to 7.5 (Fig. 1*D* and *SI Appendix*, Fig. S1).

From large-scale crystallization trials of Pb in darkness, we identified conditions at pH 5.5 that would generate yellow rod-shaped crystals. Remarkably, upon irradiation with blue light at room temperature, the crystal color transitioned to magenta, which returned to yellow/brown in darkness, revealing the presence of a photochromic bilin (Fig. 1*E*). Ultraviolet-visible (UV-vis) absorption spectra of a paste of crushed *Te*PixJ(GAF) crystals first incubated in the dark and then irradiated with blue light followed by green light confirmed photoreversibility and indicated that the crystalline Pb and Pg species had spectral properties qualitatively similar to those observed in solution (Fig. 1*F*) (6, 21), thus allowing us to examine photoconversion in crystallo.

### *Te*PixJ(GAF) Crystals Undergo Rapid Pg→Pb Thermal Reversion.

**Fig. 2.**
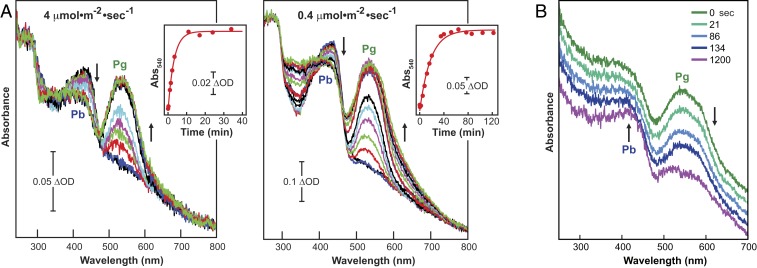
Kinetics of Pb→Pg photoconversion and Pg→Pb thermal reversion for *Te*PixJ(GAF) in crystallo. (*A*) Absorption spectra were collected at 23 °C for crushed *Te*PixJ(GAF) crystals during continuous irradiation with blue-light fluence rates of 4 μmol⋅m^−2^⋅s^−1^ (*Left*) or 0.4 μmol⋅m^−2^⋅s^−1^ (*Right*). The initial spectra (*t*_0_, black line) were collected for the dark-adapted Pb state. (*A*, *Insets*) Absorption changes at 540 nm during the photoconversion of the crystals. (*B*) Crystals of *Te*PixJ(GAF) revert thermally from Pg back to the dark-adapted Pb state. The initial spectrum (green line) was collected for Pg immediately after photoconversion with saturating blue light. Absorption spectra were then collected at the indicated incubation times in darkness at 23 °C. The spectral traces are offset for clarity. Absorption maxima are indicated.

As a first step in developing time-resolved crystallography to define *Te*PixJ(GAF) reaction intermediates, we examined the photoconversion kinetics of Pb crystals to the Pg state. As expected, higher fluences of blue light accelerated Pb→Pg photoconversion, but while the photoconversion rate constant was 0.31 (±0.02) min^−1^ at 4 μmol⋅s^−1^⋅m^−2^, it was reduced by only 5.5-fold (0.056 [±0.005] min^−1^) with 10-fold less light (0.4 μmol⋅s^−1^⋅m^−2^) (Fig. 2*A*). This discrepancy suggested that Pg→Pb thermal reversion competes with photoconversion at low fluence rates. Indeed, we detected thermal reversion of the Pg crystals upon dark incubation, with a Pb half-life estimate of several minutes at 23 °C (Figs. 1*E* and 2*B*). While this rapid thermal reversion underscored the flexibility of Pb and Pg in the crystalline state, further studies of Pb→Pg photoconversion will necessitate using high fluence rates of blue light to counteract this reaction.

### *Te*PixJ(GAF) Crystals Are Structurally Similar to Previous Crystal Forms.

**Fig. 3.**
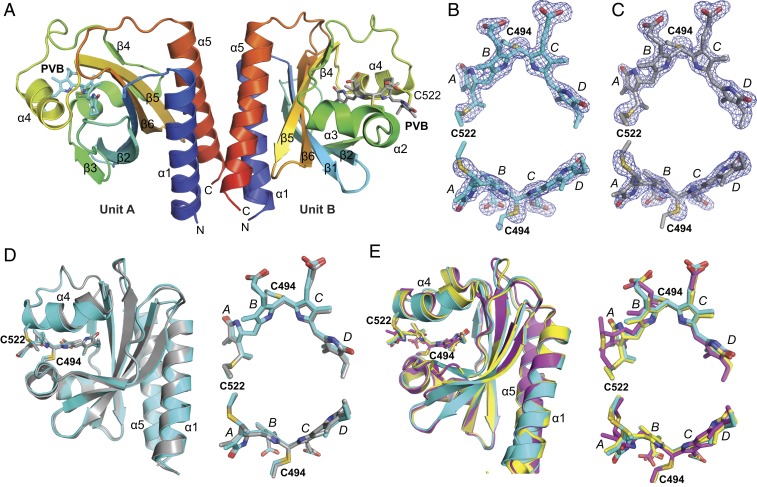
Three-dimensional structure to 1.54-Å resolution of the Pb/Pg photointerconvertible crystals from *Te*PixJ(GAF) in the *P*2_1_2_1_2_1_ space group (PDB ID code 6PRU) determined for Pb using a synchrotron X-ray source at 100 K. (*A*) Ribbon diagrams of sister GAF domains in the asymmetric unit colored as a heat map from the N terminus (blue) to the C terminus (red). The individual α-helices and β-strands are labeled for orientation. The PVB chromophore is shown as a stick model, as well as the cysteines (C494 and C522) that couple the bilin to the apoprotein via a thioether linkage. The PVB carbons in unit A (*Left*) and unit B are colored cyan and gray, respectively. Oxygens, red; nitrogens, blue; sulfurs, gold. (*B* and *C*) Refined atomic models of PVB in units A (cyan carbons) and B (gray carbons) superimposed with their omit mF_o_ − DF_c_ difference densities contoured at 3 rmsd (blue mesh). Omit difference maps were calculated after several iterations of model building and refinement but prior to modeling of PVB. C494, C522, and the 4 pyrrole rings are indicated. (*D*) Superposition and the Cα-carbons from unit A and unit B within the asymmetric unit. Units A (cyan) and B (gray) are displayed as ribbons with PVB as sticks (*Left*), along with 2 views of PVB alone after global Cα-peptide superposition (*Right*). (*E*) Superposition of PVB and the Cα-carbons from unit A (cyan) with the equivalent region in two prior crystallographic models of the GAF domain from *Te*PixJ as Pb in the *R*3_2_ and *P*4_1_2_1_2 space groups [PDB ID code 4FOF, yellow; PDB ID code 4GLQ, magenta (6), respectively]. The A to D pyrrole rings are labeled.

In an effort to generate a crystal structure of *Te*PixJ(GAF) as Pb with minimal X-ray–induced damage, we used continuous vector translation across the length of the crystals to lessen X-ray dose. These photoreversible crystals belonged to the *P*2_1_2_1_2_1_ space group and diffracted to 1.54-Å resolution at 100 K (Protein Data Bank [PDB] ID code 6PRU) (*SI Appendix*, Table S1). The asymmetric unit was occupied by 2 *Te*PixJ(GAF) molecules (designated units A and B), which dimerized in crystallo via a 4-helix bundle involving the α1- and α5-helices (Fig. 3*A*). All residues in both *Te*PixJ(GAF) molecules were visible in the electron density. Additionally, the electron density for both PVB moieties and the adjoining thioether linkages were well-resolved and continuous (Fig. 3 *B* and *C*). Superposition of the 2 molecules in the asymmetric unit showed that the main-chain atoms were highly similar throughout (root-mean-square deviation [rmsd] of 0.47 Å for all Cα-atoms) with some differences noticeable at helix α4, which appeared to subtly alter the atomic positions of the adjacent PVB moieties within their binding pockets (Fig. 3*D*).

Overall, the atomic model generated from this crystal form of *Te*PixJ(GAF) superposed well with previous Pb models generated by both X-ray crystallography (6) and 2-dimensional NMR (25) from a construction containing residues 430 to 591 followed by a 6His tag, suggesting that the core GAF domain is structurally rigid (Fig. 3*E*). Compared with the prior crystallographic models in the *R*3_2_ space group (PDB ID code 4FOF, rmsd of 0.50 Å for all Cα-atoms of residues 435 to 584) and in the *P*4_1_2_1_2 space group (PDB ID code 4GLQ, rmsd of 0.84 Å for all Cα-atoms of residues 435 to 584) (6), the most salient difference was the unique position of pyrrole ring A in the *P*4_1_2_1_2 model with accompanying differences in helix α4 (Fig. 3*E*). Interestingly, the yellow crystals in the *P*4_1_2_1_2 space group failed to change color after blue-light irradiation, implying that the chromophore environment strongly influences photoconversion. We speculate that this photoconversion defect might be caused by crowding of the chromophore by the C-terminal helix of a symmetry-related molecule in the *P*4_1_2_1_2 crystal form, which causes the disposition of the A and B pyrrole rings and helix α4. See *SI Appendix*, Fig. S2 for a comparison of the major crystalline contacts for the *P*2_1_2_1_2_1_ and *P*4_1_2_1_2 crystal forms.

Although the 2 molecules of *Te*PixJ(GAF) within the asymmetric unit were similar in structure, the environments of the chromophore differed slightly. As in previous structures (6), the B-ring propionate of unit A formed hydrogen bonds with H498 and W499. However, this contact was altered in unit B. Although the hydrogen bond with W499 was retained, H498 was displaced by R507′ from unit A in the symmetry-related molecule, which formed a salt bridge with the B-ring propionate of unit B (*SI Appendix*, Fig. S3). As judged by absorption spectra of the Pb and Pg states of crushed crystals, this ectopic salt bridge did not appear to block Pb⇋Pg interconversion. However, it could subtly constrain the C-ring propionate and subsequently impact photoconversion and thermal reversion rates, or induce differing extinction coefficients in the A and B units that might obfuscate the extent of reaction.

### Photoconversion Intermediate(s) Detected by Cryocrystallography at 150 K.

As a first attempt at assessing whether the crystal form of *Te*PixJ(GAF) would be suitable for characterizing structural intermediates along the Pb→Pg photoconversion pathway, we tested whether increased temperatures would permit the accumulation of distinct photoproducts. As shown by Yang et al. (13) for a Pfr/Pr–type bathyphytochrome, such elevated temperatures below the glass transition point can enable detection of photoproducts using X-ray diffraction by trapping the associated structural transitions. In our experiment, the temperature of a cryopreserved crystal was raised from 100 to 150 K, and a complete X-ray diffraction dataset of dark-adapted Pb was collected (*SI Appendix*, Table S1). Afterward, while still at 150 K, the same crystal was irradiated with blue light to enable the accumulation of photoproducts, and a second X-ray diffraction dataset was collected on a fresh portion of the crystal while under continuous blue-light irradiation for direct comparison with that for Pb (PDB ID code 6P58). Here, the elevated temperature for data collection did not appear to affect the quality of the electron density at the chromophore when compared with synchrotron data collection at 100 K or SFX data collection at room temperature (*SI Appendix*, Fig. S4). Although this methodology has the potential for yielding products off the Pb→Pg pathway, it should provide evidence for the capture of blue light-induced photoproducts needed for subsequent time-resolved studies. As prior data with other CBCRs demonstrated that full photoconversion requires temperatures above 200 K (16, 26), we anticipated that the blue light would generate one or more primary photoproducts and not drive rupture of the C494–C10 thioether linkage nor the complete transition to Pg.

**Fig. 4.**
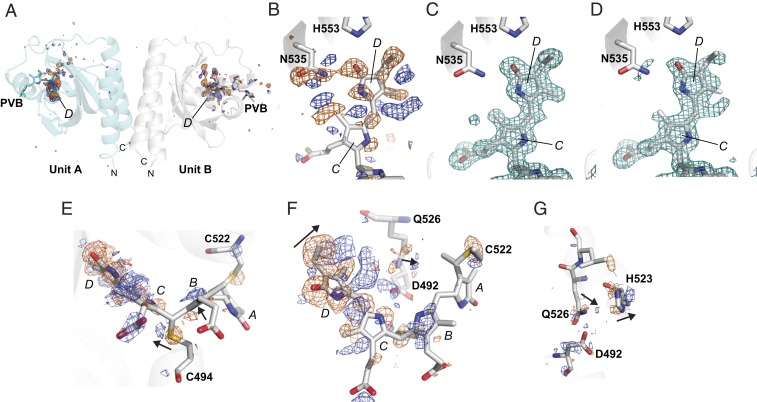
In crystallo photoexcitation of *Te*PixJ(GAF) drives conformational changes around the D pyrrole ring of PVB. X-ray diffraction datasets were collected at 150 K from a region of a crystal either maintained as Pb or following a 5-min exposure to blue light (PDB ID code 6P58). (*A*) F_o(illuminated)_ − F_o(dark)_ map to 1.5-Å resolution of illuminated–dark data for the entire asymmetric unit above/below ±4 rmsd (positive, blue; negative, orange). Large peaks were observed only at the D ring of PVB in both A and B units. (*B*) Detailed F_o_ − F_o_ map of the region surrounding the D ring in unit B above/below ±4 rmsd. These difference densities indicate that D-ring rotation and movement within the binding pocket is allowed at 150 K. (*C* and *D*) Polder chromophore omit maps at 3 rmsd (teal mesh) of the C and D pyrrole rings of PVB within unit B before (*C*) and after excitation with blue light (*D*). The Pb state of PVB is shown for comparison. (*E*–*G*) Several views of the F_o_ − F_o_ map at ±3 rmsd (positive, blue; negative, orange) focusing on regions surrounding the bilin that rearrange upon blue-light excitation at 150 K. Arrows indicate the direction of motion based on the F_o_ − F_o_ map. The A to D pyrrole rings are labeled. (*E* and *F*) Side (*E*) and top views (*F*) of PVB and neighboring amino acids. (*G*) Amino acids behind the chromophore that shift in response to PVB rotation.

Indeed, comparisons of diffraction datasets collected at 150 K from a single *Te*PixJ(GAF) crystal as Pb and then after irradiation with intense blue light clearly manifested structural changes at and around the chromophore (Fig. 4*A*). Isomorphous difference map peaks were highly localized to the D pyrrole ring for both units A and B. In fact, F_o(illuminated)_ − F_o(dark)_ maps around the D ring and the N535 side chain indicated electron density differences in excess of ±10 rmsd, which were consistent with structural changes in the bonds associated with C15 as PVB transitions toward Pg (Fig. 4*B*). Conversely, the rest of the map was featureless beyond ±4 rmsd. Polder omit maps (27) also illustrated significant differences around PVB between its Pb and photoexcited states, indicating a large-scale transition of the chromophore (Fig. 4 *C* and *D*). Taken together, it appears that blue-light excitation at 150 K allowed isomerization of the chromophore within its binding pocket at the C15 methine bridge, and relaxation of N535 into a new orientation.

Interestingly, the F_o_ − F_o_ difference peaks implied that PVB swings deeply into the binding pocket during the initial stages of photoconversion as compared with prior Pb and Pg models (6, 7). The electron density differences revealed that PVB moves away from N535 after illumination, and now is in a better position to interact with the D492 and Q526 side chains, which move slightly to accommodate the new chromophore position (Fig. 4 *B*, *D*, and *E*). The binding-pocket side chains on helix α4 also adjusted slightly to the photoproduct, based on small difference peaks at Q526, H523, and C522 (Fig. 4 *B*–*G*). Changes in the position of the thioether linkage between the C10 carbon in PVB and C494 were also consistent with sliding of the bilin (Fig. 4*D*). H523 in particular seems to shift forward toward strand β4 to make room for the chromophore photoproduct moving behind it in the binding pocket (Fig. 4 *B* and *G*).

### X-Ray Radiation Induces Rapid Decay of the Chromophore as Pg.

**Fig. 5.**
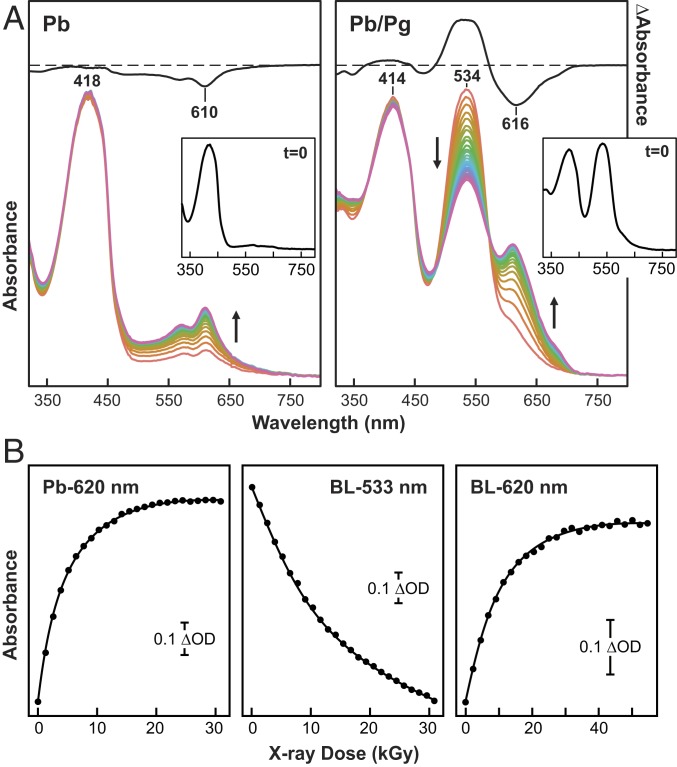
PVB chromophore in *Te*PixJ(GAF) is hypersensitive to X-ray irradiation under cryogenic conditions. (*A*) UV-vis absorption spectra collected during X-ray diffraction data acquisition at 100 K showing the appearance of new absorbing species with increasing X-ray dose (0 to >30,000 Gy) for PVB in dark-adapted (Pb) crystals versus blue light-illuminated crystals containing a mix of Pb and Pg. (*A*, *Insets*) Spectra of the Pb and the Pb/Pg mixture before X-ray exposure (*t* = 0). Initial-minus-final difference spectra are shown at half-magnitude to illustrate the extent of X-ray radiation damage and to identify the wavelength maxima and minima for the reaction products. (*B*) X-ray–mediated absorption changes of the PVB chromophore as a function of X-ray dose (kGy). Pb indicates data from a dark-adapted crystal monitored at 620 nm, and BL represents data from a blue light-illuminated crystal containing a mix of Pb and Pg that was monitored at 533 and 620 nm. Scale bars indicate the magnitude of absorption changes. The solid lines represent exponential fits to the data.

During collection of X-ray diffraction data, we noticed that the Pb crystals underwent substantial color changes, suggesting that like in our previous studies with the *Deinococcus radiodurans* Phy BphP assembled with biliverdin (28), the bilin in *Te*PixJ(GAF) degrades upon X-ray exposure. To examine this sensitivity, we measured UV-vis absorption spectra at 100 K during the collection of X-ray diffraction data at Stanford Synchrotron Radiation Lightsource BL9-2. Again, we found that the electronic state of PVB was perturbed, but surprisingly the sensitivity of Pb and Pg differed substantially. X-ray exposures of Pb crystals up to ∼50 kGy (wavelength 0.979 Å) had little effect on the absorption maximum of the crystals at 418 nm, while similar treatment of Pg crystals led to significant bleaching of its absorption maximum at 534 nm with a rate constant of 6.8 (±0.1) × 10^−6^ Gy^−1^ (Fig. 5). X-ray irradiation of either the Pb or the Pg states also generated a degradation product(s) with absorption peaks between 550 and 650 nm, which appeared with rate constants of 8.9 (±0.1) × 10^−5^ Gy^−1^ and 1.9 (±0.1) × 10^−5^ Gy^−1^, respectively (Fig. 5).

How these absorbing species were generated is not yet clear. However, comparisons of structures generated with 2.6, 0.56, and 0.26 MGy of X-ray dose (PDB ID code 6UPP) indicated density differences centered at the sulfur atoms of the thioether linkages at C494 and C522 at high dose (*SI Appendix*, Fig. S5 and Table S1), suggesting that cleavage of these bonds is one culprit. Regardless of the consequences, the emergence of these absorbing species implied that electronic-state perturbations arise during continuous X-ray irradiation of *Te*PixJ crystals, which could complicate separating bona fide reaction intermediates from species artifactually generated by X-ray exposure, especially for photo- and thermal conversion studies on Pg.

The *Te*PixJ(GAF) crystals analyzed in Fig. 5 were also assessed for diffraction quality. As shown in *SI Appendix*, Fig. S6, a crystal irradiated with near-saturating blue light (mostly Pg) retained comparable diffraction quality to the same crystal in its dark-adapted Pb state (*SI Appendix*, Fig. S6 *A*, *B*, *D*, and *E*). Additionally, we note that the ratio of the Pg and Pb maxima for the same blue light-irradiated crystal determined either by a single absorption measurement or from an average absorption measured over a 180° rotation of the crystal differed, implying anisotropy in light absorption (Fig. 5*A* and *SI Appendix*, Fig. S6*F*).

### *Te*PixJ(GAF) Is Amenable to SFX Studies.

One avenue to probe photoconversion intermediates while simultaneously minimizing X-ray–induced damage is SFX using XFEL sources. This approach would enable collection of diffraction data under biologically permissible temperatures using ultrashort X-ray pulses (femtosecond timescale), which we presume would generate diffraction patterns before the accumulation of X-ray–induced structural artifacts (24, 29). To test this approach, we applied microcrystals of *Te*PixJ(GAF) as Pb to the drop-on-tape SFX system (23). From a complete dataset of over 32,000 images collected at room temperature, a 1.55-Å model was generated (*SI Appendix*, Table S1). The high-resolution limit was chosen by conducting a series of parallel refinements with increasing high-resolution cutoffs, to find the upper-resolution limit where the data no longer improved the quality indicator *R*_free_ (*SI Appendix*, Fig. S7) (see *SI Appendix*, *Methods* for details).

**Fig. 6.**
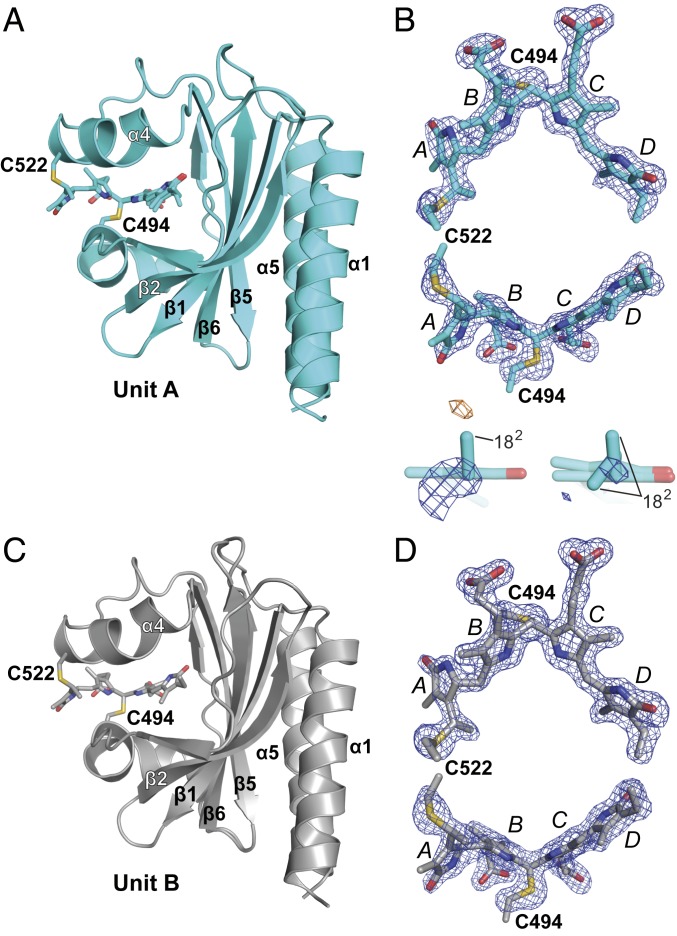
Three-dimensional structure of photointerconverible *Te*PixJ(GAF) crystals as Pb determined to 1.55-Å resolution by SFX at 297 K (PDB ID code 6PRY). (*A* and *C*) Models of (*A*) unit A and (*C*) unit B are shown in cyan and gray, respectively. The amino acids are shown in ribbon diagram with PVB modeled as sticks. (*B*) Top and side views of PVB in unit A showing the thioether linkages superimposed with mF_o_ − DF_c_ difference density (contoured at 3 rmsd calculated prior to inclusion of the PVB in the model; positive density, blue; negative density, orange). The configurations of the D ring with associated mF_o_ − DF_c_ density shown below illustrate when 1 or 2 conformations of the C18 ethyl group are modeled. (*D*) Top and side views of PVB in unit B showing the thioether linkages superimposed with mF_o_ − DF_c_ difference density as in *B*. The C494 and C552 residues that participate in the thioether linkages, pyrrole rings, and α-helices and β-strands in the polypeptide are labeled throughout.

The final SFX structure (PDB ID code 6PRY) yielded a model that included all residues of *Te*PixJ(GAF) (Fig. 6 *A* and *C*). The electron densities for the amino and carboxyl termini were more diffuse in the SFX structure than in the single-crystal synchrotron structure described above. This discrepancy could be derived from differences in data collection temperature (room temperature versus 100 K), differences in magnesium concentration (100 mM for the SFX model versus 200 mM for the synchrotron-derived model), and/or from the natural variability found in a dataset comprising thousands of crystals versus single-crystal crystallography. When compared with the Pb-state structure reported here that was modeled from conventional synchrotron X-ray diffraction, a highly congruent structure was apparent with rmsd values of 0.37 and 0.38 Å for all Cα-atoms of residues 437 to 583 of units A and B, respectively. Important for future SFX studies of illuminated crystals, the electron densities around PVB and its thioether linkages were continuous and of high quality (Fig. 6 *A* and *D*). However, while the chromophore of unit B was well-explained by a single conformation, that for unit A required a model with 2 conformations for the C18 ethyl group of pyrrole ring D, as evidenced by mF_o_ − DF_c_ difference density (Fig. 6*B*).

## Discussion

Reversible 15*Za*-to-15*Ea* isomerization is a central aspect of Phy photoconversion and appears to be conserved in most, if not all, Phys, even among those with dramatically different light–color sensitivities such as CBCRs (1, 2). However, our understanding of the Phy interconversion sequence after this transition had been limited by a lack of structural data on intermediates within the photoconversion pathways or during thermal reversion of the photoactivated state back to the dark-adapted state. In part, these deficiencies arose from the lack of highly resolved crystals needed to identify potentially subtle and/or lowly populated species. Crystal lattices also hold the potential to constrain the extent of structural transitions or rupture during transition with concomitant degradation of diffraction. These complications might be especially problematic for hairpin-containing Phys given that photoconversion is typically coupled to large conformational changes within the protein (6–11). Using the relatively simplistic photosensing module from the CBCR *Te*PixJ comprising just the GAF domain, we now show that it is possible to generate photoconvertible crystals that interconvert without compromising crystal packing or diffraction resolution. Importantly, both the absorption spectra of the blue light-irradiated crystals and the detection of similar photoproducts for both molecules within the asymmetric unit imply that nearly all of the chromoproteins in the crystal lattice undergo photoconversion.

Our cryocrystallography experiments at 150 K allowed substantial formation of photoproduct. Notably, strong electron density changes at and around the D pyrrole ring of PVB were detected for crystals irradiated with intense blue light, consistent with prior studies showing that this ring typically undergoes the most dramatic conformational changes as Pb transitions to Pg (6, 7). While the exact nature of this change is not yet clear, it was obvious that the initial photochemical transitions are centered around the 15*Za*-to-15*Ea* isomerization of PVB and rotation of the D ring followed by more subtle movements of adjacent amino acid side chains, which could stabilize the new chromophore orientation. The chromophore also appears to begin sliding in the binding pocket at 150 K, twisting toward Q526 and D492. Also notable was retention of the V-shaped configuration for PVB and electron density surrounding the thioether linkage between Cys494 and the C10 carbon of the bilin, as this D-ring twist seems to strain this bond. This retention is consistent with prior studies with another CBCR showing that formation of Pg and thus rupture of the thioether linkage at C494 might require temperatures above 200 K (16, 26). Clearly, more detailed time-resolved and temperature-scan cryocrystallographic studies are needed to resolve and validate these motions, as well as identify and temporally sequence other structural intermediates during the Pb-to-Pg transition, including when rupture of the thioether linkage at C494 occurs.

At present, we do not understand why the marginally trimmed *Te*PixJ(GAF) construction described here in the *P*2_1_2_1_2_1_ space group retains photoconversion and thermal reversion upon crystallization, unlike the previously studied noninterconvertible crystals within a *P*4_1_2_1_2 space group (6). Both crystal forms are tightly packed (42% solvent content for *P*2_1_2_1_2_1_ versus 38% for *P*4_1_2_1_2), suggesting that specific interunit contacts inhibit photoconversion. A notable difference between the 2 crystal forms is the manner in which the chromophore is held within the binding pocket, which suggests some structural microstates of Pb are photochemically inert and that interactions near the chromophore can preclude photoconversion.

One caveat to using single-crystal approaches for analyzing structural intermediates was our discovery that both the Pb and Pg states of *Te*PixJ(GAF) are particularly vulnerable to X-ray–induced alterations of the chromophore electronic structure. The absorption of Pg at the 530-nm maximum especially underwent rapid bleaching, indicative of photoproduct breakdown, with X-ray dosages typically used to collect full diffraction datasets. Furthermore, one or more orange light-absorbing species accumulated upon continued X-ray exposure, regardless of the starting photostate. The nature of these orange light-absorbing species is unclear, but they could represent PCB-type degradation products based on their bathychromic absorption relative to that of PVB. Consequently, future time-resolved studies will need to minimize/avoid these species, by using either vector translation of the X-ray beam across single crystals to minimize X-ray exposure or SFX methodologies at an XFEL or synchrotron. SFX has the advantage of being able to acquire diffraction data from singly exposed crystals at temperatures permissible for photoconversion and at femtosecond timescales presumably well before X-ray–induced degradation of the crystal can occur. Toward this goal, we showed that SFX experiments using these photoconvertible crystals of *Te*PixJ(GAF) are possible with the development of a 1.55-Å model for the Pb state based on >32,000 singly probed crystals.

In conclusion, we describe here a crystalline form of *Te*PixJ(GAF) that allows reversible photoconversion between Pb and Pg states, which should now enable the capture of Phy photoconversion intermediates via X-ray diffraction or SFX. Hopefully, this study paves the way for more detailed structural analyses of the *Te*PixJ(GAF) photoconversion at physiologically relevant temperatures, and helps connect various intermediates identified by unique spectroscopic signatures with distinct conformational states. Our success also suggests that judicious trimming of the Phy photosensory module might enable development of photoconvertible crystals for other Phy types, in particular CBCRs that typically require only the GAF domain for full photoconversion.

## Methods

All crystallization studies employed residues 435 to 584 of *Te*PixJ(GAF) that also included the C555-to-Ala substitution (6), which was preceded by a 6His tag followed by a TEV cleavage site. The 6His-TEV-*Te*PixJ(GAF) apoprotein was coexpressed in *E. coli* BL21-AI cells with the heme oxygenase (Ho1) and phycocyanobilin reductase (PcyA) from *Synechocystis* PCC6803 that direct PCB synthesis from heme, both of which were encoded within the pPL-PCB plasmid (6, 25). 6His-TEV-*Te*PixJ(GAF) was purified from cell pellets by sequential nickel-affinity and phenyl-Sepharose chromatography, followed by TEV cleavage and a subtractive nickel-affinity chromatography step (6). *Te*PixJ(GAF) crystallization screens as Pb were conducted at 20 °C in darkness by the sitting-drop vapor-diffusion method in combination with the Hampton Index or Qiagen JCSG core I screens. Well-ordered rod-shaped crystals were formed in 16 to 19% PEG 3350, 200 mM MgCl_2_, and 100 mM BisTris⋅HCl (pH 5.5). Photoconversion and thermal reversion kinetics were measured at 23 °C using the crushed crystals irradiated with 420-nm blue light for Pb or with 518-nm green light for Pg. Single-crystal X-ray diffraction datasets as Pb were collected at the GM/CA Collaborative Access Team beamline (Argonne National Laboratories) with crystals that were maintained at 100 K. Cryocrystallography datasets at 150 K were collected at the Life Sciences Collaborative Access Team 21-ID-D beamline (Argonne National Laboratories) before and during continuous irradiation with a 445-nm blue light. Merged datasets were subtracted to make F_o(illuminated)_ − F_o(dark)_ maps using Phenix isomorphous difference map tools (27) and the refined dark model for phase information. SFX data from 32,289 XFEL images of Pb crystals were collected on the MFX instrument at the Linac Coherent Light Source facility at the SLAC National Accelerator Laboratory, using the drop-on-demand method described by Fuller et al. (23). Detailed protocols can be found in *SI Appendix*, *Methods*.

### Data Availability.

X-ray diffraction datasets and associated models were deposited in the Research Collaboratory for Structural Bioinformatics (RCSB) Protein Data Bank (https://www.rcsb.org) under PDB ID codes 6P58, 6PRU, 6PRY, and 6UPP.

## Supplementary Material

Supplementary File
