# Supplementary material for: Photoreversible interconversion of a phytochrome photosensory module in the crystalline state
Source: Proc Natl Acad Sci U S A. 2019 Dec 18;117(1):300–7. doi: 10.1073/pnas.1912041116 (PMC6955287; doi:10.1073/pnas.1912041116)
Supplement: Supplementary File [file pnas.1912041116.sapp.pdf]

Supplemental Information Appendix

Photoreversible Interconversion of a Phytochrome Photosensory Module in the Crystalline State

E. Sethe Burgie *et al.*

Methods

Protein Expression and Purification. All crystallization studies employed residues 435-584 of *Te*PixJ(GAF) that also included the Cys555 to Ala substitution to discourage aggregation (1). This coding region was inserted by Gibson assembly (2) into a modified pBAD vector (3), onto which was appended the coding regions for N-terminal 6His tag followed by a tobacco etch virus (TEV) cleavage site (MGSSHHHHHHSSENLYFQG) to the 5' end. The 6His-TEV-*Te*PixJ(GAF) apoprotein was co-expressed in *E. coli* BL21-AI cells with the heme oxygenase (Ho1) and phycocyanobilin reductase (PcyA) from *Synechocystis* PCC6803 that direct PCB synthesis from heme, both of which were encoded within the pPL-PCB plasmid (1, 4). Cells were first grown at 37°C to 1 OD_{600nm} in Terrific Broth containing 1 mM $MgSO_4$ and 250 µM 5-aminolevulinic acid, and then incubated for an additional one hr at 16°C. Isopropyl β-D-1-thiogalactopyranoside was added to 1 mM to induce the expression of the PCB biosynthetic genes, which was followed one hr later by addition of 0.2% L-arabinose to induce expression the 6His-*Te*PixJ(GAF) polypeptide (5). Cultures were grown overnight at 16°C, harvested by centrifugation, and immediately frozen at liquid nitrogen temperatures. Unless otherwise noted, all culture growths, purification of 6His-*Te*PixJ(GAF) as Pb, and subsequent manipulations of the samples were performed at 0-4°C in darkness or under dim green safelights.

6His-TEV-*Te*PixJ(GAF)-containing cell pellets were disrupted by sonication into lysis buffer (50 mM HEPES-NaOH (pH 7.8), 500 mM NaCl, 0.05% Tween-20, 1 mM 2–mercaptoethanol, 1 mM phenylmethylsulfonyl fluoride, 10% glycerol, and 1 Pierce EDTA-free complete protease inhibitor tablet per liter) also containing 30 mM imidazole as described (1). Following clarification at 35,000 x *g,* the chromoprotein was enriched from the supernatant by nickel-affinity chromatography using nitrilotriacetic acid (NTA) beads (Qiagen ~20 mL) equilibrated in lysis buffer containing 30 mM imidazole. The beads were washed with 10 column volumes of the same buffer followed by several column volumes of lysis buffer supplemented with 300 mM imidazole to elute 6His-TEV-*Te*PixJ(GAF). The eluate was exchanged into 75 mM ammonium sulfate, 150 mM imidazole, 10 mM 2–mercaptoethanol, and 5 mM HEPES-NaOH (pH 7.8), concentrated to ~1 mL with 10-kDa MWCO centrifuge filter, and then fractionated isocratically with a 20 mL phenyl-sepharose HP column (GE) equilibrated with the same buffer. The 6His-TEV tag in the 6His-TEV-*Te*PixJ(GAF)-containing fractions was excised by overnight

incubation with TEV protease. The sample was exchanged into extraction buffer using a 10-kDa MWCO centrifuge filter, and passed through a subtractive nickel-NTA column equilibrated with the same buffer to remove the tag, TEV protease, and other contaminants. Cleaved *Te*PixJ(GAF) in the flow through was concentrated with a 10-kDa MWCO centrifuge filter and subjected to gel filtration with a 24-mL Superdex 75 column (GE) equilibrated with 10 mM HEPES-NaOH (pH 7.8), 50 mM NaCl, and 0.3 mM Tris(2-caroxylethyl)phosphine for buffer exchange and further enrichment. Purified samples were concentrated to 25 mg•ml^{-1} with 10-kDa MWCO centrifuge filters and flash frozen as ~30 µl pellets in liquid nitrogen.

Crystallization of *Te*PixJ(GAF). Suitable crystallization conditions were developed at 20°C in darkness by the sitting drop vapor diffusion method set up with a TTP Mosquito crystallization robot in combination with the Hampton Index or Qiagen JCSG core I screens. Trials combined 200 nL of *Te*PixJ(GAF) at 25 mg•mL^{-1} with 200 nL of reservoir solution. Well-ordered rod-shaped crystals formed when the chromoprotein solution was mixed with an equal volume of reservoir solution containing 16-19% PEG 3350, 200 mM $MgCl_2$, and 100 mM BisTris-HCl (pH 5.5). Crystals analyzed by synchrotron X-ray sources were flash cooled in liquid nitrogen after equilibration with reservoir solution formulated to include 20% ethylene glycol. Where indicated, crystals were irradiated for 20 sec immediately prior to flash freezing with 420-nm (FWHM ~ 16 nm) light provided by a Thorlabs M420F2 LED powered to 25% capacity by a DC2200 LED driver (Thor Labs) from a distance of 5 mm. Microcrystal slurries for analysis by SFX were generated by layering without mixing 130 µL of a 25 mg•mL^{-1} *Te*PixJ(GAF) solution onto a 130 µL precipitant solution containing 34% PEG 3350, 200 mM $MgCl_2$, and 200 mM BisTris-HCl (pH 5.5). Microcrystals appeared within seconds, and were fully formed within one day.

Spectroscopic Analyses. UV-vis absorption spectra of *Te*PixJ(GAF) in solution or as crushed crystals were recorded at 25ºC and 23ºC, respectively, with a Cary 60 UV-Vis spectrophotometer (Agilent). Prior to recording the solution spectra, the protein was exchanged into 100 mM $MgCl_2$ and either 100 mM BisTris-HCl (pH 5.5 at 25 ºC), 100 mM BisTris-HCl (pH 6.5 at 25 ºC), or 100 mM HEPES-NaOH (pH 7.5 at 25 ºC). Spectra were collected for samples either adapted to darkness (Pb), immediately upon photoconversion to the Pg state with saturating blue light as above at 19.5 µmol•m^{-2}•sec^{-1} for 90 sec, or after the samples were photoconverted back to Pb with saturating green light (λ_{max} ~ 518 nm) provided by a Superbrightleds G11 LED. To limit oxidation of C494, green light was applied to the sample immediately after the absorption spectrum of Pg was collected.

Absorption spectra of crystals were generated from SFX-suitable microcrystals prepared as above. To concentrate the crystals and limit the solution state of *Te*PixJ(GAF), they were

collected by two rounds of centrifugation for 10-15 sec at 1,600 x *g*, removal of the supernatant, and resuspension of the crystals into a precipitant solution containing 20% PEG 3350, 100 mM MgCl2, and 100 mM BisTris-HCl (pH 5.5). The final crystal paste was crushed between glass cover slides sealed with vacuum grease, and placed in the spectrometer beam to measure Pb and Pg absorption as above. To collect spectra of the Pg state *in crystallo*, the sample was pretreated with 420 nm light (FWHM ~ 16 nm) for 45 sec at 100 μmol•m-^{2}•sec^{-1}. The Pb state was then regenerated for spectra collection as above.

Photoconversion and thermal reversion kinetics of *Te*PixJ(GAF) crystals were measured at 23ºC using the crushed crystals. Photoconversion was assessed in a non-continuous manner by illuminating the crystalline samples with either 0.4 or 4 μmol•m^{-2}•sec^{-1} blue light (420 nm) as above for various times followed by spectrophotometric measurement. Thermal reversion was conducted by driving the sample to steady state as Pg with 4 μmol•m^{-2}•sec^{-1} blue light, and allowing the sample to relax back to Pb at 23ºC in darkness with continued absorption measurements.

UV-Vis absorption spectra were acquired at 100 K from single crystals during collection of X-ray diffraction data at beamline 9-2 of the Stanford Synchrotron Radiation Lightsource using a custom single crystal microspectrophotometer that employs a QE65000 spectrometer (6). Optimal positioning of the crystals was determined by collecting spectra as a function of Phi-angle similar to Orville et al. (7). Absorption spectra were then collected just prior to X-ray diffraction data collection and then consecutively after every 50 frames during the 180º rotation of the crystals. As a reference, raw intensity counts were collected minus the crystal (Reference) and without the irradiation beam (Dark). Absorption spectra of the crystals were calculated as follows: Absorption = log[(Reference – Dark)/(counts with sample present – Dark)]. Fits of Pb to Pg photoconversion as a function of time and X-ray damage as a function of X-ray dose were calculated in R using Raddose-3D (8) to calculate X-ray dose. The data were fit to a single exponential based on $Abs = \Delta Abs \bullet e^{-kx} + Abs_{\infty}$, where Abs is absorbance, k is a rate constant, x is either time or X-ray dose, and Abs_{∞} is the absorbance at steady state.

X-ray Diffraction Data Collection and Structural Determination. X-ray diffraction data were collected by single-crystal diffraction at a synchrotron source or by serial femtosecond diffraction at the LCLS XFEL. For the former, datasets were collected at the GM/CA Collaborative Access Team beamline (Argonne National Laboratories Advanced Photon Source) and SSRL BL-9-2 with crystals that were maintained at 100 K. For the former, the data were indexed and integrated with XDS (9), and scaled with aimless (10) using the autoPROC pipeline (11).

XFEL data were collected at the MFX instrument (12) of the LCLS facility (SLAC National Accelerator Lab, Menlo Park, CA) using X-ray pulses of <40 fsec length at an energy of 9.5 keV, and with an average pulse power of 2.3 mJ and a beam size of 3 μm (FWHM), obtained by compound refractive lenses. Sample was delivered (~2.5-3 mL•min^{-1}) from suspensions of microcrystals (1.2 x 10^7 crystals•mL^{-1}) by the Drop-On-Demand method (13). The 2.5-nL droplets were deposited on a Kapton tape traveling at 300 mm•sec^{-}1 and transversed a helium enclosure for 0.8 sec before the X-ray pulse. Diffraction data were detected at a rate of 10 Hz on a Rayonix MX 170HS detector with 2x2 binning at a distance of 93.7 mm from the interaction point.

SFX data from 32,289 images were processed using *cctbx.xfel* and DIALS as described (14, 15), and were indexed using an initial target unit cell of a=42.28 Å, b=61.24 Å, c=116.45 Å, α=β=γ=90° and space group $P2_12_12_1$. After joint refinement of the Rayonix detector model against 3000 crystal models, a new average unit cell of a=44.79 Å, b=61.42 Å, c=116.48 Å, α=β=γ=90 was calculated, which was used to re-index and integrate the entire dataset. The images were merged using *cxi.merge* within the *cctbx.xfel* package (16). A per-image resolution cutoff (based on resolution-binned I/σ(I) estimates) was applied to each image, each of which was then post-refined by applying a partiality correction to inflate the unmerged partially-recorded reflections from the still images to their full equivalent values. The final dataset was merged to 1.55 Å.

Initial phases were obtained by molecular replacement using Phaser (17) from the Phenix graphical user interface (18). The search model was derived from the prior model of *Te*PixJ(GAF) spanning residues 443-578 (PDB ID 4FOF (1)). To avoid bias, the PVB chromophore was omitted from the search model and the amino acids within the binding pocket were modeled as alanines (*i.e.*, Y463, I490, N492, C494, F495, H498, W499, Y503, R507, Q509, C522, H523, Q526, L527, L530, V532, N535, V537, L549, I551, and H553). Using the Phenix GUI, initial automated refinement after molecular replacement included default settings plus rigid body refinement and simulated annealing (17). Afterwards, *Te*PixJ(GAF) models were rebuilt manually using Coot (19), and refined with Phenix (18). Structure validation was conducted with Molprobity (20). Superpositions were generated and illustrations were prepared with the PyMOL Molecular Graphics System (http://www.pymol.org/). Diffraction images were prepared for Figure S6 using Adxv (http://www.scripps.edu/tainer/arvai/adxv.html).

Establishment of the High-Resolution Limit for SFX Refinement. To assess the high-resolution limit used for refinement of the SFX datasets, we conducted a series of parallel refinements based on the concept of paired refinements promoted by Karplus and Diederichs (21). Specifically, eight perturbed starting models were generated from an intermediately refined model by resetting the isotropic B-factors to 30 Å and applying random shifts to the x,y,z coordinates (max 0.25 Å protein / 0.15 Å solvent, with average RMSD shifts of 0.144 Å and 0.087

Å for the protein and solvent). The R_{free} for these models increased from 0.197 to around 0.295. The 8 models were then individually refined with 10 different high-resolution cutoffs between 1.5 Å and 2.1 Å. R-factors computed for the resulting 80 refined models at each of the resolution cutoffs allowed comparison with common sets of reflections similar as with the paired refinement. Thus, all R_{free} values calculated using a specific high-resolution limit of the data could be compared among all models that were refined at that or higher resolution limits to find the high-resolution limit of model refinement that minimized R_{free}. This was repeated using several different automated refinement protocols, which led to a general trend of refinement of data to 1.55-Å resolution with the lowest R_{free} values. Reasonable I/σ(I) values were found in the highest resolution shell (1.5), although the $CC_{1/2}$ values appeared to be low (0.012). This was consistent with the observations of Bublitz *et al.* (22) who also found that including higher resolution SFX data helped refinements despite low $CC_{1/2}$ values.

Cryo-crystallography at 150 K. Data were collected at the Life Sciences Collaborative Access Team 21-ID-D beamline (Argonne National Laboratories Advanced Photon Source). Large crystals of *Te*PixJ(GAF) were produced using the SFX protocol, cryopreserved in loops, and mounted onto the goniometer with the cryostat set at 100 K. The cryostat was then adjusted to 150 K for the remaining steps. For the dark-adapted Pb state, 180 degrees of diffraction data were collected using a 10 µm x 10 µm X-ray beam on a non-irradiated region of the rod-shaped crystal. The crystal was then illuminated for 5 min with a 445-nm 7.1 W SOLIS light-emitting diode (Thor Labs), which was powered at 4.0 A by a DC2200 LED driver (Thor Labs). The crystal was then translated 50 µm down its long axis to a fresh region, and then 180 degrees of diffraction data were collected with continuous LED illumination using the same protocol as the dark dataset. The data were integrated with DIALS (23), and scaled and merged with aimless (10) using the xia2 pipeline (24). Identical sets of R-free flags were added in Phenix (18). Merged datasets were subtracted to make $F_{o(illuminated)}$-$F_{o(dark)}$ maps using the Phenix isomorphous difference map tools and the refined dark model for phase information. Polder omit maps that exclude bulk solvent in the omitted region of the chromophore were generated with Phenix (25).

References

1. Burgie ES, Walker JM, Phillips GN, Jr., & Vierstra RD (2013) A photo-labile thioether linkage to phycoviolobilin provides the foundation for the blue/green photocycles in DXCF-cyanobacteriochromes. *Structure* 21(1):88-97.
2. Gibson DG*, et al.* (2009) Enzymatic assembly of DNA molecules up to several hundred kilobases. *Nat. Methods* 6(5):343-345.
3. Burgie ES*, et al.* (2017) Photosensing and thermosensing by phytochrome B require both proximal and distal allosteric features within the dimeric photoreceptor. *Sci. Rep.* 7(1):13648.
4. Cornilescu CC*, et al.* (2014) Dynamic structural changes underpin photoconversion of a blue/green cyanobacteriochrome between its dark and photoactivated states. *J. Biol. Chem.* 289(5):3055-3065.
5. Gambetta GA & Lagarias JC (2001) Genetic engineering of phytochrome biosynthesis in bacteria. *Proc. Natl. Acad. Sci. USA* 98(19):10566-10571.
6. Cohen AE, Doukov T, & Soltis MS (2016) UV-visible absorption spectroscopy enhanced X-ray crystallography at synchrotron and X-ray free electron laser sources. *Protein Pept. Lett.* 23(3):283-290.
7. Orville AM*, et al.* (2011) Correlated single-crystal electronic absorption spectroscopy and X-ray crystallography at NSLS beamline X26-C. *J. Synchrotron Radiat.* 18(Pt 3):358-366.
8. Zeldin OB, Gerstel M, & Garman EF (2013) Optimizing the spatial distribution of dose in X-ray macromolecular crystallography. *J. Synchrotron Radiat.* 20(Pt 1):49-57.
9. Kabsch W (2010) XDS. *Acta Crystallogr. D Biol. Crystallogr.* 66(Pt 2):125-132.
10. Evans PR & Murshudov GN (2013) How good are my data and what is the resolution? *Acta Crystallogr. D Biol. Crystallogr.* 69(Pt 7):1204-1214.
11. Vonrhein C*, et al.* (2011) Data processing and analysis with the autoPROC toolbox. *Acta Crystallogr. D Biol. Crystallogr.* 67(Pt 4):293-302.
12. Sierra RG*, et al.* (2019) The macromolecular femtosecond crystallography instrument at the linac coherent light source. *J. Synchrotron Radiat.* 26(Pt 2):346-357.
13. Fuller FD*, et al.* (2017) Drop-on-demand sample delivery for studying biocatalysts in action at X-ray free-electron lasers. *Nat. Methods* 14(4):443-449.
14. Brewster AS*, et al.* (2016) Processing XFEL date with cctbx.xfel and DIALS. *Comp. Crystallo. Newslett.* 7:32-53.
15. Brewster AS*, et al.* (2018) Improving signal strength in serial crystallography with DIALS geometry refinement. *Acta Crystallogr. D Struct. Biol.* 74(Pt 9):877-894.
16. Sauter NK (2015) XFEL diffraction: developing processing methods to optimize data quality. *J. Synchrotron Radiat.* 22(2):239-248.

17. McCoy A*, et al.* (2007) Phaser crystallographic software. *J. Appl. Crystallogr.* 40(4):658-674.
18. Adams P*, et al.* (2010) PHENIX: a comprehensive Python-based system for macromolecular structure solution. *Acta Crystallogr. D-Biol. Crystallogr.* 66:213-221.
19. Emsley P & Cowtan K (2004) COOT: model-building tools for molecular graphics. *Acta Crystallogr. D Biol. Crystallogr.* 60(Pt 12 Pt 1):2126-2132.
20. Chen V*, et al.* (2010) MolProbity: all-atom structure validation for macromolecular crystallography. *Acta Crystallogr. D Biol. Crystallogr.* 66(Pt 1):12-21.
21. Karplus PA & Diederichs K (2012) Linking crystallographic model and data quality. *Science* 336(6084):1030-1033.
22. Bublitz M*, et al.* (2015) Structural studies of P-type ATPase-ligand complexes using an X-ray free-electron laser. *IUCrJ* 2(Pt 4):409-420.
23. Winter G*, et al.* (2018) DIALS: implementation and evaluation of a new integration package. *Acta Crystallogr. D Struct. Biol.* 74(Pt 2):85-97.
24. Winter G (2010) xia2: an expert system for macromolecular crystallography data reduction. *J. Appl. Crystallogr.* 43:186-190.
25. Liebschner D*, et al.* (2017) Polder maps: improving OMIT maps by excluding bulk solvent. *Acta Crystallogr. D Struct. Biol.* 73(Pt 2):148-157.
26. Hattne J*, et al.* (2014) Accurate macromolecular structures using minimal measurements from X-ray free-electron lasers. *Nat. Methods* 11(5):545-548.

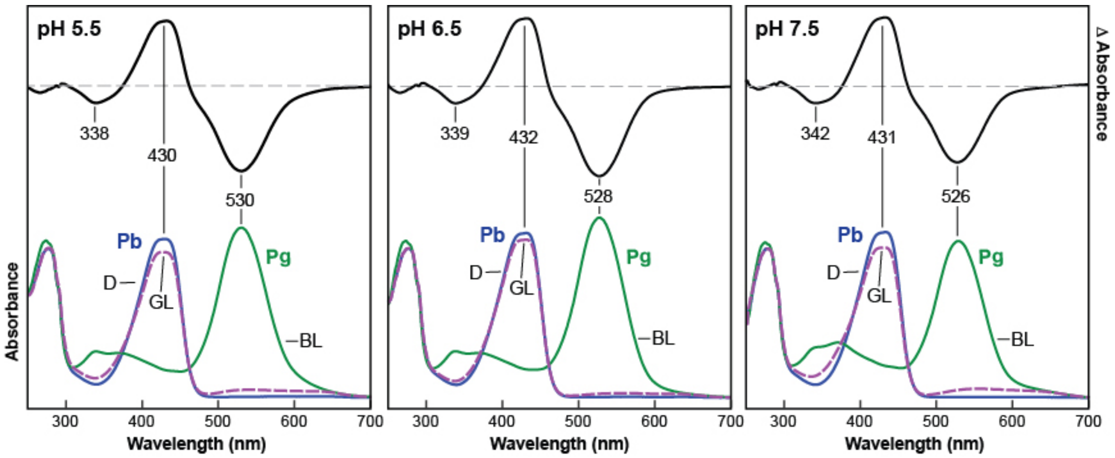

Figure S1. Solution absorption spectra of *Te*PixJ(GAF) at 25 °C as Pb and Pg at a pH range of 5.5 to 7.5. The absorption spectra for Pb (blue lines) were recorded for dark-adapted samples whereas those for Pg (green lines) were recorded after a saturating irradiation with blue light (BL). The purple dashed line identifies the absorption spectra of the photocycled Pb product generated by irradiating Pg with green light (GL). Dark-adapted Pb minus Pg difference spectra (black line) are shown above at half magnitude. The wavelengths of difference peak minima or maxima are indicated.

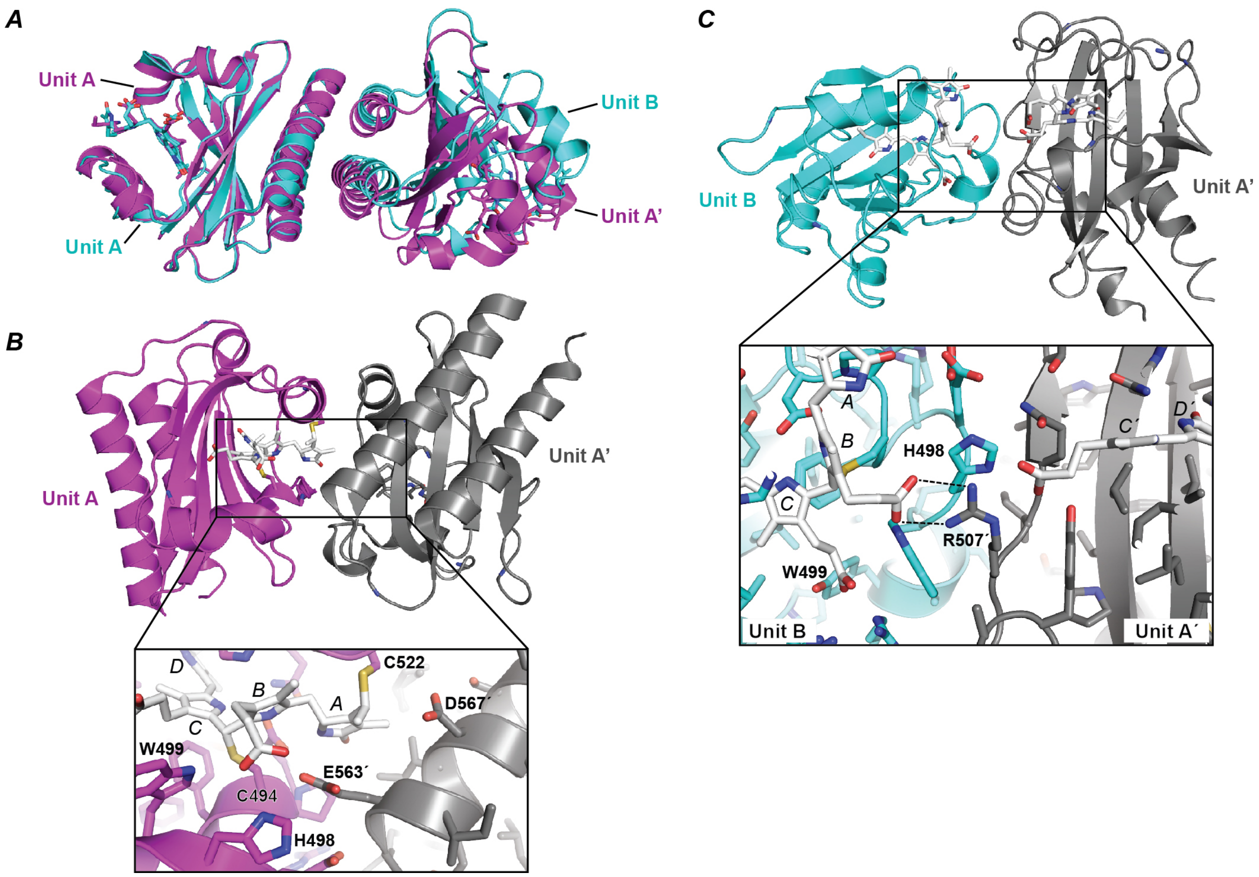

Figure S2. Crystal contact comparisons of photoconvertible *Te*PixJ(GAF) crystals in the $P2_12_12_1$ space group versus nonphotoconvertible crystals in the $P4_12_12$ space group (PDB ID: 4GLQ). Although the *Te*PixJ GAF domain is monomeric in solution, it forms head-to-head dimers with similar contact points in both the $P2_12_12_1$ and $P4_12_12$ crystals. **(*A*)** Superposition of the structural models obtained from crystals in the $P2_12_12_1$ (cyan) and $P4_12_12$ (magenta) space groups. **(*B*)** Close-up of the contacts between asymmetric units in the $P4_12_12$ space group showing a major contact surface between the binding pocket of one protomer (Unit A, magenta) and the C-terminal helix of a second protomer (Unit A′, gray). This contact disposes the A and B pyrrole rings through direct association with residues E563′ and D567′ of the C-terminal α helix. **(*C*)** Close-up of the contacts between asymmetric units in the $P2_12_12_1$ space group showing major contacts near the chromophore-binding pocket of Unit B (cyan) and the chromophore binding pocket of Unit A′ (gray). These contacts do not significantly dispose the chromophore. See also Fig. S3. For (***B***) and (***C***) the affected chromophore carbons are colored white for contrast. Both models were generated from data collected at a synchrotron X-ray source.

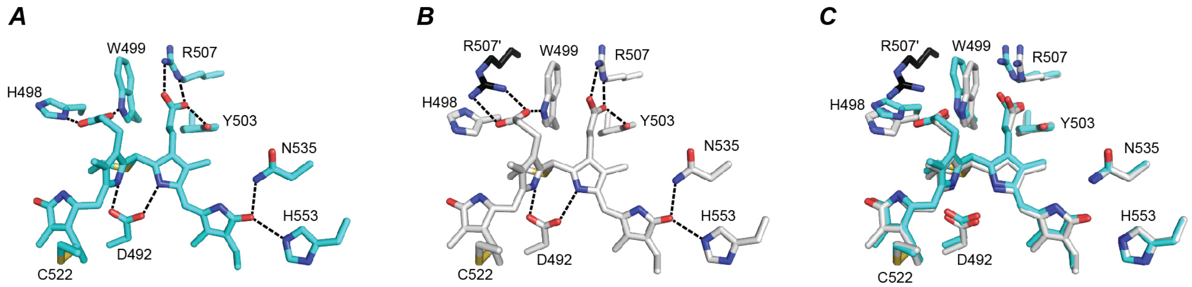

Figure S3. 3D structure of the bilin-binding pocket in Pb/Pg photointerconvertible crystals in the $P2_12_12_1$ space group from *Te*PixJ(GAF) showing an ectopic salt-bridge between the B-ring propionate of Unit B and an arginine from a crystal symmetry mate. (***A***) Hydrogen-bonding network of Unit A (cyan carbons). (***B***) Hydrogen-bonding network of Unit B (gray carbons) that includes an ectopic interaction with R507′ (black carbons) from Unit A of a symmetry related molecule (Unit A'). (***C***) Superposition of Units A and B, highlighting the binding pocket differences. The model for Pb was generated using data collected from a synchrotron X-ray source.

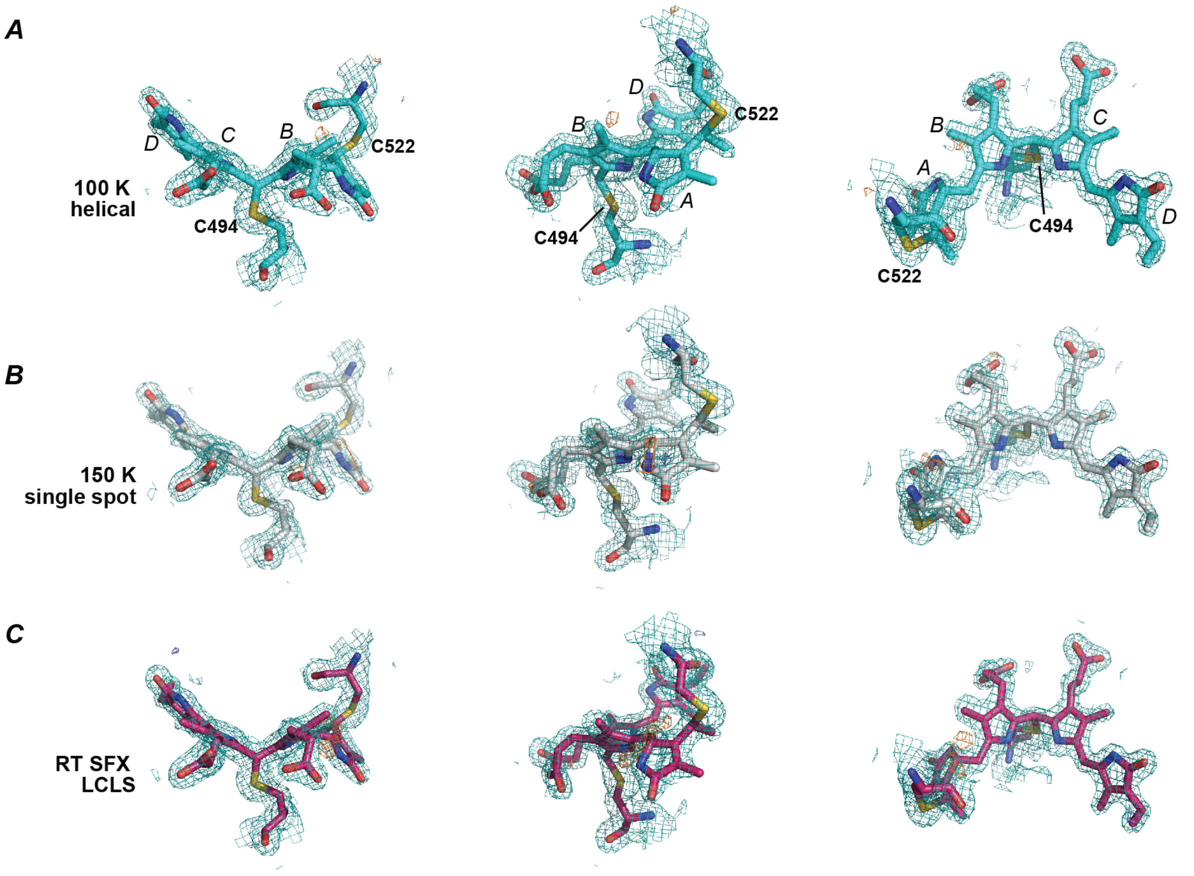

Figure S4. Comparison of chromophore electron density between Pb datasets collected either at 100 K by helical vector translation to minimize radiation damage, at a single position of a crystal at 150 K, or at room temperature by SFX (***C***). (***A-C***) Different viewing angles for PVB superimposed with its associated electron density for the structure generated at 100 K by helical vector translation (***A***, cyan), a single crystal at 150 K (***B***, gray), or at room temperature (RT) by SFX (***C***, magenta). mF_o-DF_c difference density was contoured at 3 RMSD (positive, blue; negative, orange). $2mF_o$-DF_c was shown at 1 RMSD (teal).

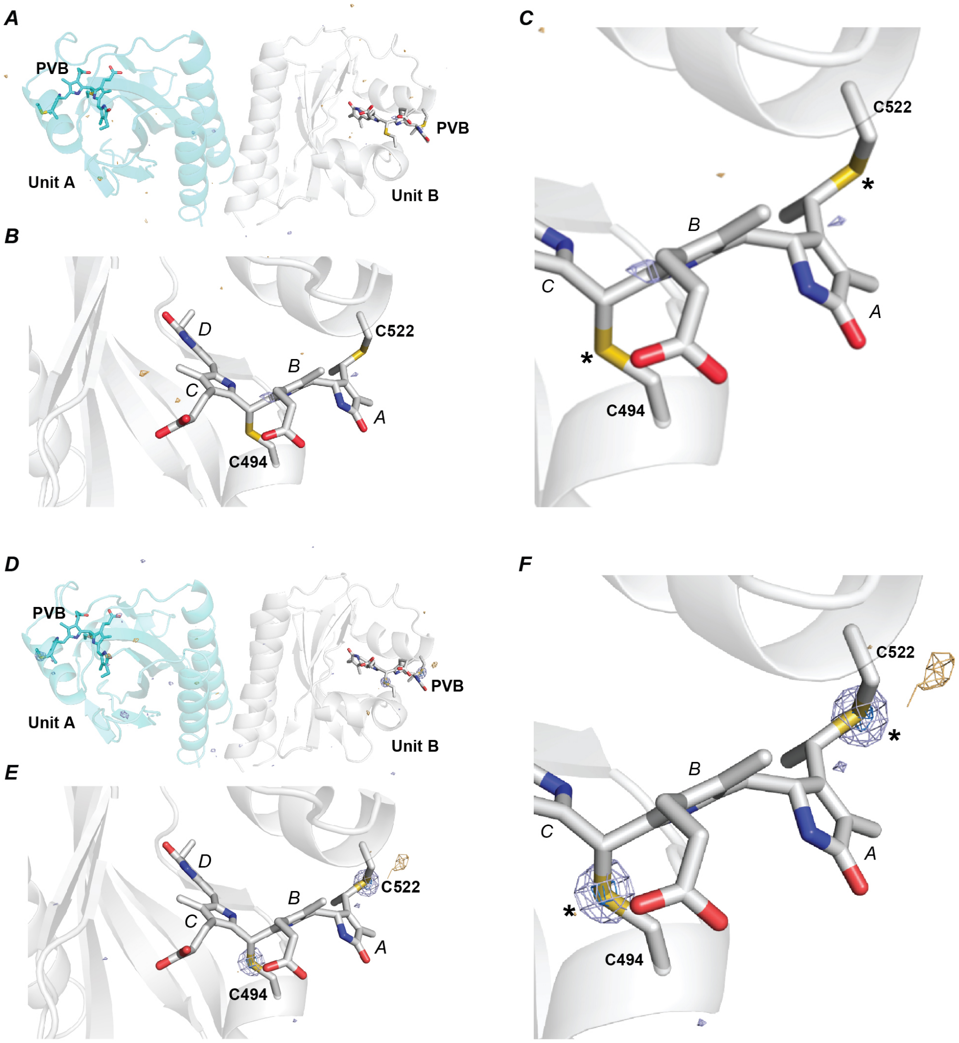

Figure S5. Sensitivity of the bilin within *Te*PixJ(GAF) to X-ray damage. (***A-C***) Various views of the F_o-F_o map comparing changes in the electron density surrounding the chromophore generated at 0.26 versus 0.56 MGy X-ray doses for a single crystal as Pb. (***D-F***) Various views of the F_o-F_o map comparing changes in the electron density surrounding the chromophore generated at 0.26 versus 2.6 MGy X-ray doses for a single crystal as Pb. (***A*** and ***D***) View of the asymmetric Unit. (***B, C, E,*** and ***F***) Close up views of PVB. Shown are F_o-F_o comparisons at +/- 4 RMSD (blue/light orange) and +/- 8 RMSD (dark blue/orange), which localized X-ray-induced alterations around the thioether linkages at C494 and C522 in high X-ray doses. The A-D pyrrole rings are labeled.

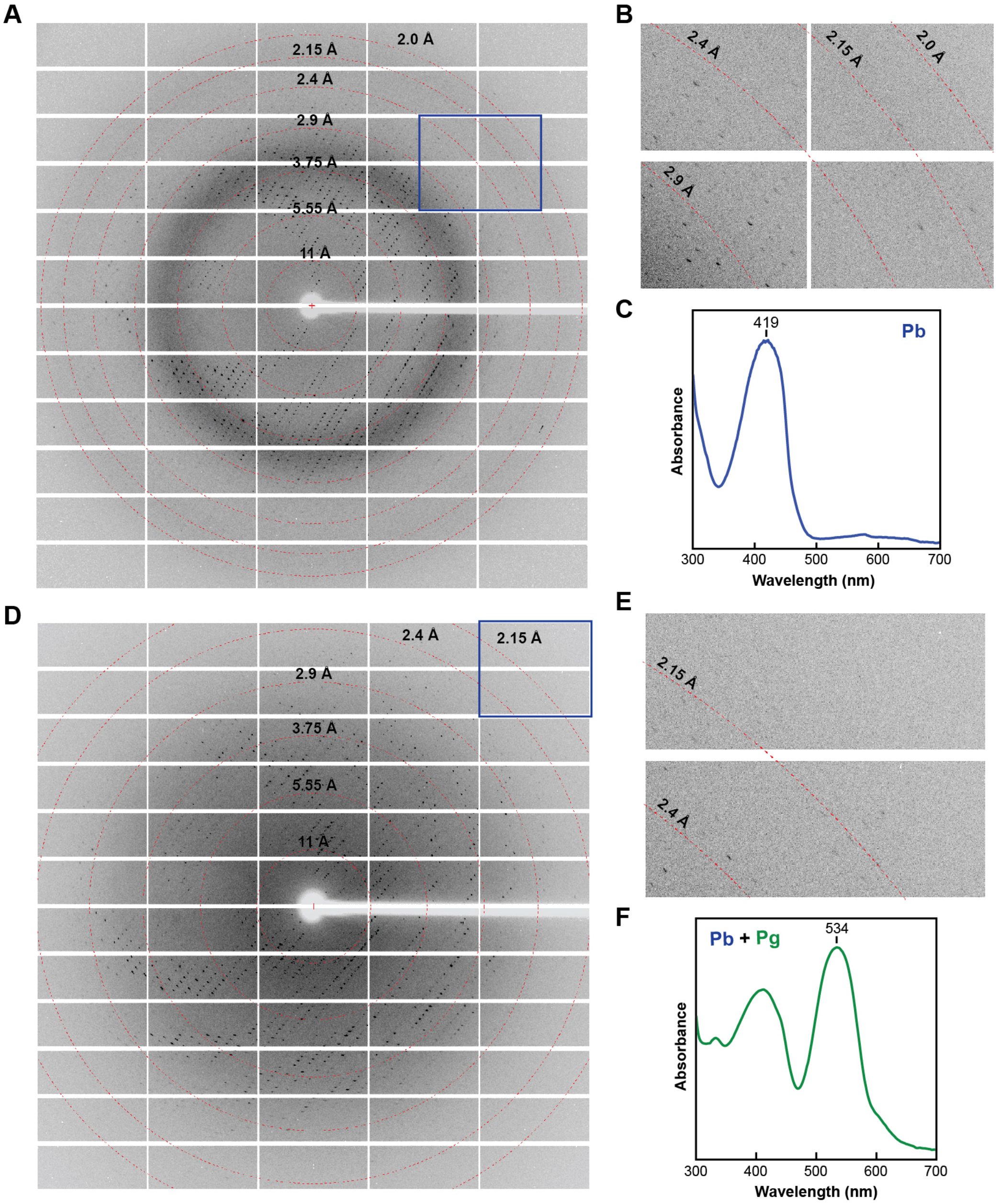

Figure S6. Diffraction quality of *Te*PixJ(GAF) crystals highlighted in Figure 5 in the dark-adapted Pb state (***A-C***), or after reaching steady-state levels of Pg after blue-light irradiation at room temperature (***D-F***). (***A*** and ***D***) Diffraction data for crystals merged from a 3° sweep of data. (***B*** *and* ***E***) Magnification of a high-resolution section of the diffraction data (delineated with a blue box). (***C*** and ***F***) Average UV-Vis absorption spectrum of the crystal collected over a 180° rotation of a crystal.

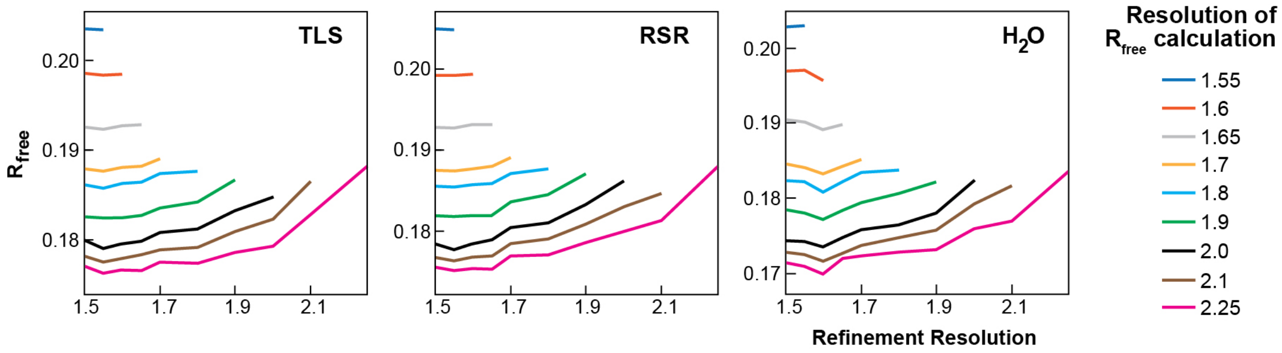

Figure S7. Methods used to define the resolution limit for refinement for the SFX data. Shown are the analyses of model improvement as a function of resolution for the X-ray diffraction data. The average R_{free} from 8 different starting models was plotted as a function of the resolution of model refinement. Individual lines represent the high-resolution cutoff used for the comparison R_{free} calculation. Plots show the results from refinements conducted using three different scripted protocols: XYZ, B, and translation-libration-screw (TLS); XYZ, B, TLS, RSR and automated water update (H_2O); and XYZ, B, TLS and real-space refinement (RSR)
